# Perfectionism as a predictor of physician burnout

**DOI:** 10.1186/s12913-022-08785-7

**Published:** 2022-11-28

**Authors:** Sarah R. Martin, Michelle A. Fortier, Theodore W. Heyming, Kyle Ahn, Whitney Nichols, Charles Golden, Haleh Saadat, Zeev N. Kain

**Affiliations:** 1 Department of Anesthesiology and Perioperative Care, University of California, Irvine, 505 S. Main Street, Suite 940, Orange, CA 92868 USA; 2 Center On Stress & Health, University of California, Irvine, Orange, CA USA; 3 Children’s Hospital of Orange County, Orange, CA USA; 4 Sue & Bill Gross School of Nursing, University of California, Irvine, Irvine, CA USA; 5 Department of Emergency Medicine, University of California, Irvine, Irvine, CA USA; 6 Frank H Netter MD School of Medicine at, Quinnipiac University, North Haven, USA

**Keywords:** Burnout, Perfectionism, Physicians

## Abstract

**Background:**

Burnout is common among physicians and has detrimental effects on patient care and physician health. Recent editorials call attention to perfectionism in medicine; however, no studies to date have examined the effect of perfectionism on burnout in physicians practicing in the United States. This study examined associations among demographics, perfectionism and personality traits, and burnout among practicing physicians.

**Methods:**

This cross-sectional study included general pediatric and pediatric sub-specialist physicians. Out of the 152 physicians contacted, 69 enrolled (Mean_age_ = 44.16 ± 9.98; 61% female). Emotional exhaustion, depersonalization, and personal accomplishment burnout were assessed via the Maslach Burnout Inventory. Validated instruments were used to measure personality and perfectionism. Data were analyzed using linear regression models.

**Results:**

Across physicians assessed, 42% reported either high emotional exhaustion burnout or depersonalization burnout. High self-critical perfectionism uniquely predicted both high emotional exhaustion burnout (*B* = 0.55, 95%CI 0.25–0.85) and depersonalization burnout (*B* = 0.18, 95%CI 0.05–0.31). Low conscientiousness (*B* = -6.12; 95%CI, -10.95- -1.28) predicted higher emotional exhaustion burnout and low agreeableness (*B* = -3.20, 95%CI -5.93- -0.46) predicted higher depersonalization burnout.

**Conclusions:**

Perfectionism is understudied among physicians and the current findings suggest that addressing system and individual-level factors that encourage perfectionism is warranted and may reduce risk for physician burnout.

## Background

Burnout is highly prevalent among practicing physicians and across specialties. Sample data from the United States indicate that 45–50% of physicians endorse high levels of burnout, with internal medicine and emergency medicine reporting the highest rates of burnout [1, 2]. This clinical phenomena consists of work-related emotional exhaustion, an impersonal attitude, and a decreased sense of competence [3]. Because burnout has detrimental effects on quality of care, patient satisfaction, and physician mental health and health behaviors [3–7], identifying predictors associated with physician burnout may help identify those at risk for burnout and prevent negative outcomes. Previous physician burnout literature has primarily focused on demographic and occupational-related factors associated with burnout, with results indicating that physicians who are female, younger, have less experience and higher workloads are more likely to experience burnout [1, 2, 4, 8, 9]. Other work found that high neuroticism [10, 11], low agreeableness [10, 11], and low conscientiousness [11] personality traits may be associated with higher burnout in physicians. Physician burnout may also be challenged by the COVID-19 pandemic, with recent data indicating burnout prevalence rates up to 75% [12–14].

In recent years, the role of perfectionism in medical professional wellness has been raised by several investigators [15, 16]. Perfectionism is a multidimensional trait exemplified by overly high and unreasonable standards, strivings for flawlessness, and criticism of oneself and others [17], which is different from striving for high, albeit attainable standards [18, 19]. Perfectionism is common among physicians and within the culture of medicine [16, 20] and broader literature links perfectionism to a range of negative mental health outcomes [21]. Recent editorials call attention to the prevalence of perfectionism in medicine and the potential role of perfectionism in physician burnout [15, 16]. Data from medical student and nurse samples indicate that perfectionism is associated with psychological distress and poor job satisfaction [22, 23], but no studies to date have examined the effect of perfectionism on burnout in physicians practicing in the United States.

The current cross-sectional study aimed to characterize burnout among physicians in a children’s hospital network during the COVID-19 pandemic and examine the effects of physician demographics as well as perfectionism and personality traits on different dimensions of burnout. Based on recent editorials on perfectionism [15, 16], we hypothesized that higher perfectionism would be associated with higher burnout. Consistent with previous physician studies [10, 11], we expected that neuroticism, agreeableness, and conscientiousness would be associated with burnout.

## Methods

### Participants

This cross-sectional study included a sample of 69 general pediatricians and pediatric sub-specialists (hereinafter referred to as “physicians”) within a single children’s hospital network in Southern California. Inclusion criteria included being an attending physician affiliated with the children’s hospital care network. Nurse practitioners and physician assistants were excluded. Out of the 152 physicians contacted, 69 enrolled (Mean _age_ = 44.16 ± 9.98; 61% female). The sample included general pediatricians (20%), emergency medicine physicians (33%), hospitalists (3%), and other subspecialists (43%; e.g., urology, gastroenterology).

### Measures

#### Demographics

Participants self-reported sex, race, ethnicity, pediatric specialty, years of experience, and average number of patient’s seen per day.

#### Personality

The Big Five Inventory [24], a well-validated measure [25, 26], includes 44-items and assessed personality factors, including openness (“*comes up with new ideas*”; sample ω = 0.78), conscientiousness (“*a reliable worker*”; sample ω = 0.84), extraversion (“*is talkative*”; sample ω = 0.89), agreeableness (“*is helpful and unselfish with others*”; sample ω = 0.73), and neuroticism (“*worries a lot*”; sample ω = 0.87).

#### Perfectionism

The 45-item Big Three Perfectionism Scale [17] questionnaire measured three global perfectionism factors: rigid perfectionism (reflects insistence that personal performance is without errors; “*I have a strong need to be perfect*”; sample ω 0.93), self-critical perfectionism (tendency for negative reactions to mistakes, self-criticism, assuming others demand perfection; “*I judge myself harshly when I don’t do something perfectly*”; ω = 0.88), and narcissistic perfectionism (beliefs that one is superior, expected to be perfect, expect others to be perfect; “*It is important to me that other people do things perfectly*”; sample ω = 0.90). This questionnaire that has demonstrated acceptable reliability and validity [27].

### Primary outcome

#### Burnout

Maslach Burnout Inventory – Human Services Survey (MBI-HSS for Medical providers) is a widely accepted, validated burnout measure that assesses emotional exhaustion (e.g., “*I feel emotionally drained from my work*”), depersonalization (e.g., “*I’ve become more callous toward people since I took this job*”), and personal accomplishment (e.g., “*I feel I’m positively influencing other people’s lives through my work*”) dimensions of burnout in human, healthcare service workers [3]. The authors of the MBI-HSS recommend utilizing continuous burnout scores when examining predictors or outcomes of burnout, but *low*, *moderate* and *high*population norms can be used to characterize levels of burnout across samples [3]. Sample Omega values for Emotional Exhaustion, Depersonalization and Personal Accomplishment were 0.92, 0.82, and 0.85 respectively.

### Procedures

A research team member recruited physicians via an email sent to the entire medical staff and provided a copy of the study information sheet, which included a description of the study, risks, benefits, and alternatives to participation. Informed consent was obtained from all individual participants included in the study. Interested physicians completed consent and study questionnaires using a REDCap survey link. Out of the 152 physicians emailed, 69 responded and were all eligible (response rate of 45.4%). Participants completed study questionnaires at one timepoint between August 2020 and February 2021. Study procedures were approved under expedited review by the Institutional Review Boards of Children’s Hospital Orange County (January 7, 2021/ 1,502,878–8) and all study procedures were performed in accordance with the relevant guidelines and regulations.

### Statistical analyses

Descriptive statistics were calculated to characterize the sample and missing data were handled by measure guidelines or, if guidelines were not available, participant-level means were imputed for scales with no more than 10% of data missing. Three participants did not complete any items on the perfectionism, personality, or burnout measures. Burnout and perfectionism data were skewed and did not pass normality tests. To characterize the sample based on published burnout norms [3], we examined the proportion of the sample that reported low, moderate, and high levels of emotional exhaustion, depersonalization, and personal accomplishment burnout. Given that other burnout studies characterize high burnout as reporting either high emotional exhaustion or depersonalization [1, 28], we also examined this metric of burnout in the current sample.

For the primary analyses, consistent with measure guidelines [3], we utilized continuous burnout scores as the dependent variable. Specifically, non-parametric mean difference and correlational analyses were conducted to examine associations between burnout and demographic and physician characteristic variables. Variables that were significantly associated with emotional exhaustion, depersonalization, or personal accomplishment burnout were included in subsequent linear regression analyses. Spearman’s rank correlational analyses were conducted to assess associations among independent variables (i.e., physician characteristics). Multiple linear regressions analyses examined unique effects of the identified physician characteristics on the three dimensions of burnout. Regression assumptions were tested, and data passed normality of residuals, homogeneity of variance, and collinearity tests. If independent variables within a regression model were strongly correlated (*r*_*s*_ > 0.50), only one variable was entered into the model. The strong association between neuroticism and self-critical perfectionism (*r*_*s*_ = 0.55, *p* < 0.001) met this criterion so only self-critical perfectionism was included in the regression model. Threshold for statistical significance was *p*< 0.05. Statistical analyses were conducted using IBM SPSS Statistics for Windows, Version 27.0. Armonk, NY:IBM Corp [29]. The datasets analyzed during the current study are available from the corresponding author on reasonable request.

## Results

Table 1 includes sample demographic descriptive statistics including frequencies, means, and standard deviations. A total of 69 physicians enrolled (response rate of 45.4%). Based on normative data [3], 28% of physicians endorsed high levels of emotional exhaustion burnout, 25% endorsed high levels of depersonalization burnout, and 10% reported high personal accomplishment burnout, which is reflected by low personal accomplishment scores (Table 2). Across physicians, 42% reported either high emotional exhaustion *or* high depersonalization burnout.

**Table 1:** Sample characteristics

Variable	Statistic
	**Mean (SD)**
Age	44.16 (9.98)
Years of Experience	13.08 (1.26)
	**Median (IQR)**
Patients Per Day	20.00 (15.00)
General Pediatricians	20.00 (5.00)
Emergency Medicine	30.00 (7.50)
Hospitalist	10.00 (0.00)
Other Pediatric Specialists	15.00 (11.50)
	**N (%)**
Specialties	
General Pediatricians	14 (20)
Emergency Medicine	23 (33)
Hospitalist	2 (3)
Other Subspecialists ^a^	30 (43)
Gender ^b^	
Female	42 (61)
Male	27 (39)
Ethnicity	
Latinx, Hispanic	5 (7)
Non-Latinx, Hispanic	62 (90)
Prefer Not Answer	1 (0.4)
Race	
African American, Black	1 (0.4)
Asian, Pacific Islander	27 (39)
White	33 (48)
Multi-Racial	7 (12)
Prefer Not Answer	1 (1)

^a^Subspecialists include surgery, urology, gastroenterology, pulmonology, neurology, endocrinology, and otolaryngology

^b^The Gender question included non-binary response options in addition to female and male

**Table 2:** Burnout descriptive results

	Median (IQR)	Low^a^ (%)	Moderate^a^ (%)	High^a^ (%)
Emotional Exhaustion (*n* = 61)	18.00 (19.50)	52	20	28
Depersonalization (*n* = 61)	5.00 (8.50)	52	23	25
Personal Accomplishment (*n* = 61)	43.00 (8.00)	70	20	10

^a^Low, moderate, high burnout cutoffs are as follows: Emotional Exhaustion: 0–18, 19–26, ≥ 27; Depersonalization: 0–5, 6–9, ≥ 10; Personal Accomplishment: > 40, 34–39, ≤ 33

### Bivariate analysis: predictors of burnout (Table 3)

**Table 3:** Correlations between physician characteristics and burnout

**Variable**	**Mean (SD)**	**Burnout**		
		**Emotional Exhaustion**	**Depersonalization**	**Personal Accomplishment**
Perfectionism				
Rigid	25.28 (8.63)	0.05	0.05	0.20
Self-Critical	35.09 (9.04)	0.44**	0.39**	-0.20
Narcissistic	33.59 (9.16)	0.17	0.22	-0.10
Personality				
Extraversion	3.29 (.84)	-0.10	-0.08	0.33*
Agreeableness	4.11 (.46)	-0.23	-0.40**	0.35**
Conscientiousness	4.18 (.55)	-0.36**	-0.29*	0.60**
Neuroticism	2.47 (.77)	0.57**	0.28*	-0.36**
Openness	3.54 (.55)	-0.16	-0.15	0.39**

^*^p < 0.05; **p < 0.01

Correlational analyses indicated that self-critical perfectionism and extraversion, agreeableness, conscientiousness, and neuroticism personality traits were significantly associated with burnout (Table 3). Emergency medicine (EM) physicians reported significantly lower emotional exhaustion burnout (14.45 ± 9.91) than non-EM physicians (21.51 ± 12.08; *Z* = -2.24, *p* = 0.025).

### Regression analyses: unique predictors of burnout (Table 4).

**Table 4:** Effects of physician characteristics on burnout

Emotional Exhaustion	R^2^_*Adj*_	B (95% CI)	SE	t	p	f^2^
Model	0.31				< .001	0.45
EM Specialty (0 = No, 1 = Yes)		-5.47 (-11.09–0.15)	2.80	-1.95	.06	0.25
Conscientiousness		-6.12 (-10.95- -1.28)	2.41	-2.54	.01	0.39
Self-Critical Perfectionism		0.55 (0.25–0.85)	0.15	3.67	< .001	0.72
***Depersonalization***						
Model	0.21				.002	0.27
Agreeableness		-3.20 (-5.93- -0.46)	1.36	-2.35	.02	0.39
Conscientiousness		-1.22 (-3.61–1.18)	1.19	-1.02	.31	0.14
Openness		0.73 (-1.52–2.99)	1.13	0.65	.52	0.09
Self-Critical Perfectionism		0.18 (0.05–0.31)	0.06	2.86	.006	0.52
***Personal Accomplishment***						
Model	0.30				< .001	0.43
Extraversion		0.99 (-0.71–2.69)	0.85	1.17	.25	0.15
Agreeableness		0.53 (-2.58–3.65)	1.55	0.34	.73	0.04
Conscientiousness		3.87 (1.10–6.63)	1.38	2.80	.007	0.45
Neuroticism		-1.38 (-3.21–0.45)	0.91	-1.51	.14	0.20
Openness		0.99 (-1.51–3.50)	1.25	0.80	.43	0.10

*EM* Indicates emergency medicine

To control for confounding variables, we next conducted a series of multiple regression models.

#### Emotional exhaustion burnout

The overall model including EM specialty, conscientiousness, and self-critical perfectionism accounted for 31% of the variance in emotional exhaustion (*F* (3, 56) = 9.45, *p* < 0.001). Higher self-critical perfectionism and lower conscientiousness significantly predicted higher emotional exhaustion burnout (Table 4).

#### Depersonalization burnout

This model accounted for 21% of the variance in depersonalization burnout (*F* (4, 56) = 4.80, *p* = 0.002). Lower agreeableness and higher self- critical perfectionism significantly predicted higher depersonalization burnout (Table 4).

#### Personal accomplishment burnout

Conscientiousness was the only variable to uniquely predict higher personal accomplishment, or lower burnout, with the overall model accounting for 30% of the variance in burnout (*F* (5, 56) = 5.86, *p* < 0.001).

## Discussion

The current study aimed to characterize burnout among physicians in a children’s hospital network during the COVID-19 pandemic and examine the effects of physician demographics, perfectionism and personality traits on different dimensions of burnout. Under the conditions of this study, 42% of physicians endorsed either high emotional exhaustion or high depersonalization burnout. Specific personality types, such as perfectionism, were identified to have unique effects on each of the burnout dimensions.

Current results suggest that perfectionism is an important risk factor for physician burnout. Perfectionistic characteristics are highly common among physicians and the expectation for perfect performance is inherent in the culture of medicine [16, 20]. Although striving for high levels of performance and minimizing errors may protect against medical errors and ensure quality patient care [16, 30], our results suggest that perfectionism may contribute to both emotional exhaustion and depersonalization dimensions of burnout. Consistent with our findings, data from medical students in Korea [31] as well as broader literature outside of medicine further support this link between perfectionism and burnout [32, 33]. In the current study, the self-critical perfectionism scale emerged as the only perfectionism scale associated with emotional exhaustion and depersonalization burnout. The self-critical perfectionism scale reflects three dimensions of perfectionism: a tendency to be highly self-critical of less than perfect performance [34], uncertainty about the quality of one’s own performance [35], and the expectation that others demand perfection or socially-prescribed perfectionism [17, 36]. These unique perfectionistic characteristics may result in behaviors or maladaptive responses that contribute to burnout. Specifically, self-critical perfectionism may promote a strong sense of self-responsibility, distress, and shame related to experienced or anticipated failure to achieve perfect performance, and significant effort towards meeting assumed high or unrealistic standards of others [15, 36].

To our knowledge, only one other study that included Italian workers, has assessed perfectionism and burnout during the COVID-19 pandemic and found similar associations between perfectionism in non-health care providers and burnout. Physicians with higher self-critical perfectionism may be particularly challenged during the pandemic. The clinical care changes, uncertainty, and increased risk associated with COVID-19 may place added stress on individuals that are highly critical of and uncertain about their performance [4, 13]. Efforts to uphold these perceived internal and external high standards during a pandemic may be particularly challenging and may help explain the unique effect of self-critical perfectionism on dimensions of burnout that reflect emotional and physical depletion and detachment from peers and patients [4].

The proportion of physicians in the current sample that endorsed either high emotional exhaustion or depersonalization burnout is both lower [1, 28, 37] and higher [9, 38, 39] than pre-COVID-19 burnout rates from diverse physician samples. Burnout rates in this study must be considered in the context of the COVID-19 pandemic, which presented a myriad of challenges (e.g., burden of new care protocols and risks to personal and family safety) that may have contributed to burnout in the current sample which focuses on physicians [40]. Recent work, although limited, has reported considerable variability in burnout among health care professionals during the COVID-19 pandemic, with global rates of burnout ranging from 13 to 76% [14]. Prevalence of physician burnout in the current study was lower than average rates of burnout during COVID-19 among US health care professionals [13], but higher than burnout reported by US EM physicians [41] and pediatric neurosurgeons [42] within three months of the US COVID-19 pandemic. We assessed burnout five to eleven months into the pandemic. The prolonged exposure to the pandemic as well as political and social justice stressors experienced by physicians, their patients and families may explain higher rates in our sample.

Consistent with adult physician burnout literature [10, 11], conscientiousness and agreeableness personality traits were associated with lower burnout; however, significant effects varied across the different dimensions of burnout. Conscientiousness includes facets of organization and productiveness and this trait was associated with lower emotional exhaustion and personal accomplishment burnout, which suggests that it may be protective against feeling emotionally depleted and incompetent in the context of COVID-19-related changes in clinical care. Similarly, compassion, trust and respectfulness traits associated with agreeableness may have influenced the degree of interpersonal detachment and contributed to lower levels of depersonalization burnout while navigating clinical and personal stressors during the pandemic.

The current study findings should be considered in the context of methodological limitations. While the participation response rate was comparable to other physician survey studies, [38, 41, 42] the convenience sampling and the sample size is a limitation. We omitted a post hoc power analysis as these analyses can provide an inaccurate estimate of power. [43] Although the current study detected significant associations with moderate to large effect sizes, additional prospective studies with larger sample sizes are needed to confirm current results. The time frame for which this sample was collected coincides with a particular time period of the pandemic, locally and internationally. The effects of the COVID-19 pandemic during this time period likely would have a large effect on individual responses. Collecting more data and comparing the current time period with that of the study would likely introduce more variability and as such, the data would not address our research questions for this paper. Recruitment from a single children’s hospital may have implications for selection bias and external validity. The timing of survey completion as well as factors not assessed including marital status or COVID-19-related concerns for safety or workplace or family stressors, may have affected burnout in our sample.

In the current study, 42% of physicians endorsed high emotional exhaustion or depersonalization burnout during the COVID-19 pandemic. Results suggest that self-critical perfectionism and certain personality traits and may predict physician burnout. Although identifying specific interventions to address perfectionism and its effect on burnout goes beyond the scope of this study, the self-critical dimension of perfectionism reflects both beliefs about one’s own performance and also the expectation that others demand perfection. Addressing multilevel factors that may promote perfectionism is warranted. On a system level, demanding and reinforcing error-free performance may set unrealistic expectations for physicians, encourage a pervasive culture of unattainable perfectionism, and may contribute to physician depression, shame, and burnout [15, 20]. On an individual level, screening physicians for perfectionism may help identify those at risk for poorer outcomes and in need of support. Given that the negative effects of perfectionism on burnout has been reported in medical students [31], it may be beneficial to implement screening early on in training to support the development of more adaptive behaviors. Interventions aimed at promoting resilience and enhancing appropriate coping skills (e.g., mindfulness, cognitive interventions) represent promising approaches to minimize burnout among physicians [44].

## Data Availability

The dataset generated during and analyzed during the current study are available from the corresponding author [ZK] on reasonable request.
